# Contrast transthoracic echocardiography in a patient with drug‐induced hypersensitivity syndrome with right atrial thrombus: A case report and mini review

**DOI:** 10.1002/pdi3.52

**Published:** 2024-04-23

**Authors:** Hui‐ru Zhu, Ting‐ting Ran, Xu Zhu, Xiaojuan Ji

**Affiliations:** ^1^ Department of Ultrasound Children's Hospital of Chongqing Medical University National Clinical Research Center for Child Health and Disorders Ministry of Education Key Laboratory of Child Development and Disorders Chongqing China; ^2^ Chongqing Key Laboratory of Pediatrics Chongqing China; ^3^ Department of Ultrasound Chongqing General Hospital Chongqing China

## 1 INTRODUCTION

Contrast transthoracic echocardiography (cTTE) is a technique based on transthoracic echocardiography (TTE), in which an acoustic contrast agent is injected through a peripheral vein to enhance the visualization of the right cardiac system, thus detecting the right‐to‐left shunt (RLS) of blood flow. In 1968, Gramiak et al. first injected an oscillating mixture of indocyanine blue‐green and physiological saline directly into the right heart via cardiac catheterization, which created the precedent of cTTE. In recent years, with the development of science and technology and the improvement of medical imaging equipment, cTTE has provided a new method for clinical diagnosis and treatment of cardiac and pulmonary vascular diseases. At the same time, compared to cardiac computed tomography (CT) and MR imaging techniques, the contrast agents used in cTTE are nonradioactive, less allergenic than those used in CT and MR, and can be excreted from the body through the alveoli, with minimal impact on renal function and high reproducibility.^1^ Currently, cTTE is commonly used to detect patent foramen ovale (PFO).

Drug‐induced hypersensitivity syndrome (DIHS) is a complex adverse drug reaction characterized by systemic involvement, notably with the reactivation of human herpesvirus 6. This syndrome manifests after prolonged exposure to specific drugs, encompassing a protracted incubation period, and carries with it a potentially fatal prognosis.^2^ If it is not treated in time, it will lead to significant mortality. Thrombosis is not one of the common complications of DIHS. Here, we will report a case of right atrial thrombus in a child with DIHS detected by cTTE.

## 2 CASE PRESENTATION

An 1‐years‐old female infant presented with a constellation of symptoms, including rash, cough, and fever. Approximately 6 weeks prior, the child had been hospitalized at a local medical facility, where she was diagnosed with acute respiratory distress syndrome (poisoning) and severe pneumonia after accidentally ingesting electric mosquito repellent (pyrethroid insecticide). Over the subsequent 3 weeks, the infant progressively developed a distinctive red rash that initially manifested around the waist and then extended to encompass the trunk, extremities, and face, eventually becoming partially confluent. Following hospitalization, she received a comprehensive treatment regimen, inclusive of mechanical ventilation support, antibiotic therapy (comprising ceftazidime and meropenem), epinephrine administration, and intravenous sodium methylprednisolone succinate.

The pediatric patient was evaluated in our respiratory ward and was presented with a complex medical condition encompassing cooccurring with DIHS. In response to this diagnosis, an anti‐infective therapeutic regimen comprising amikacin and metronidazole was promptly initiated. Subsequently, she was transferred to the Pediatric Intensive Care Unit due to the emergence of severe diarrhea and a pronounced electrolyte disturbance, notably potassium ion measuring 5.58 mmol/L, sodium ion at 121 mmol/L, and chloride ion at 94.6 mmol/L.

On March 24, a cardiac auscultation identified the presence of a heart murmur, prompting the attending physician to order a bedside echocardiogram, which unveiled the presence of moderately echogenic masses within the patient's right atrium. Subsequently, between March 24 and April 21, the patient underwent seven ultrasound examinations including three cTTE.

In the ultrasound report dated March 31, it was mentioned that a long, occupying lesion, characterized by homogeneous internal echogenicity, predominantly hyperechoic, with augmented marginal echogenicity and well‐defined borders, was observed in the long‐axis view of the inferior vena cava near its junction with the right atrium. Remarkably, this lesion exhibited oscillatory motion in sync with the cardiac cycle and occupied a space measuring approximately 26.0 mm by 9.3 mm within the right arterial cavity.

**FIGURE 1:**
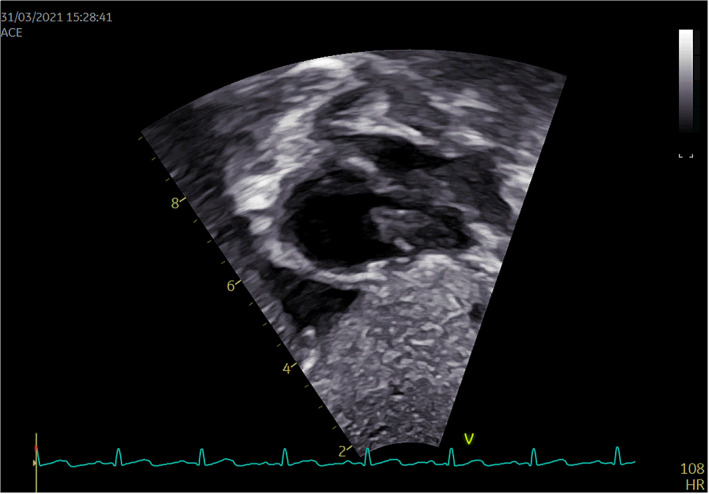
Pretreatment ultrasound 2D dynamic images.

**FIGURE 2:**
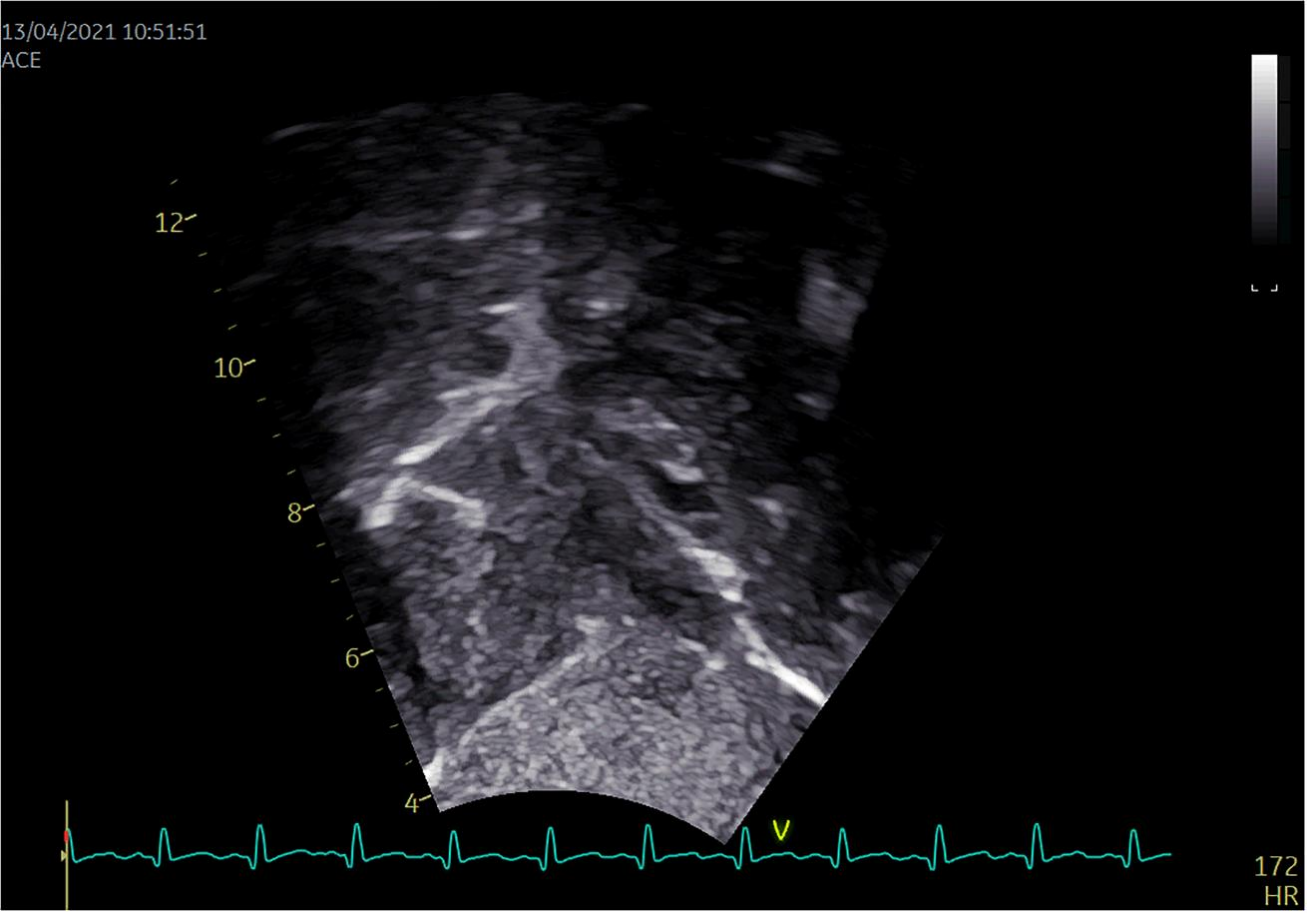
Pretreatment contrast transthoracic echocardiography image.

**FIGURE 3:**
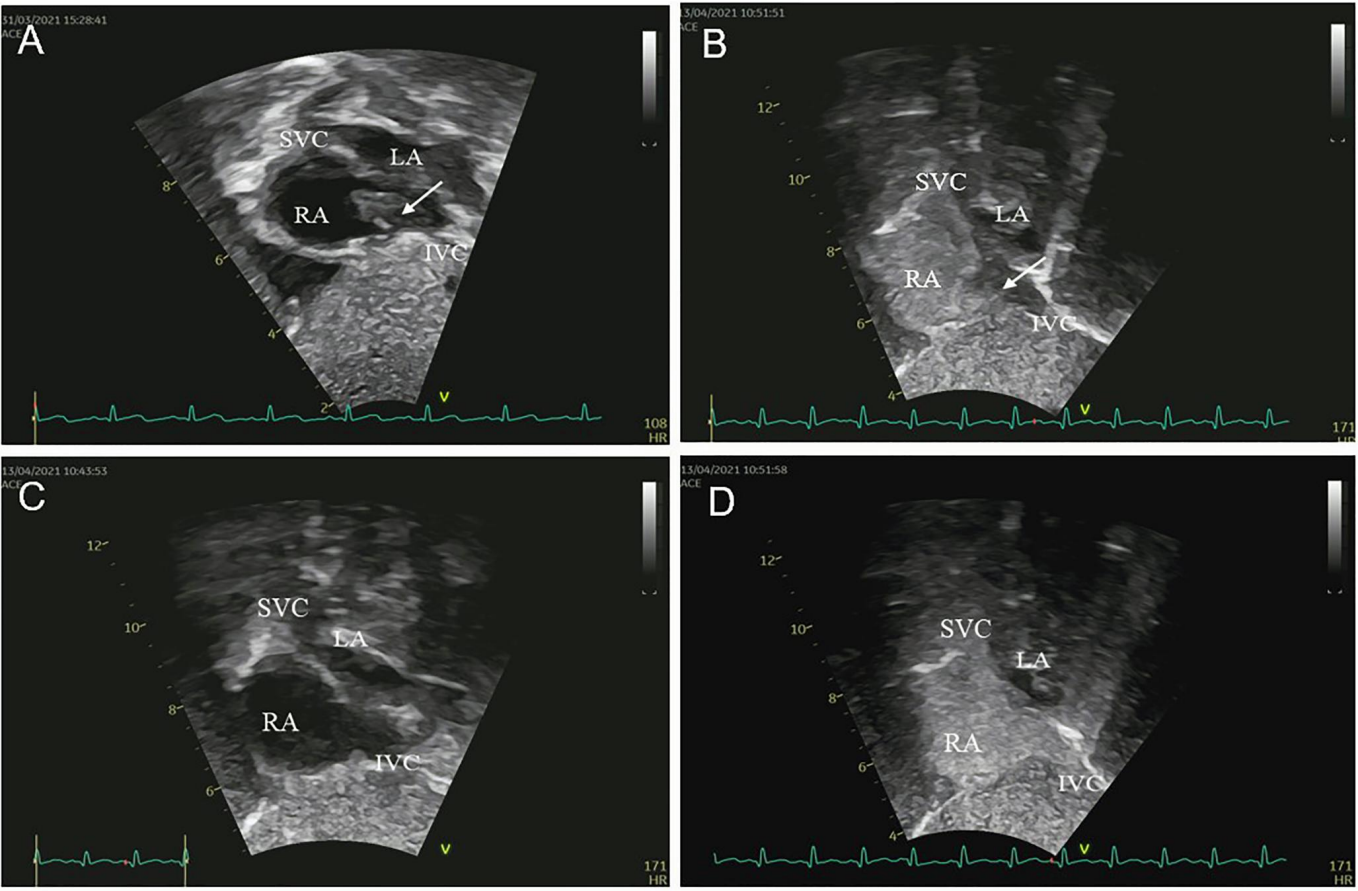
Pre and post‐treatment ultrasound images. (A) Pretreatment ultrasound 2D image. (B) Pretreatment cTTE image. (C) Post‐treatment ultrasound 2D image. (D) Post‐treatment cTTE image. cTTE, contrast transthoracic echocardiography; LA, left atrium; RA, right atrium; SVC, superior vena cava.

**FIGURE 4:**
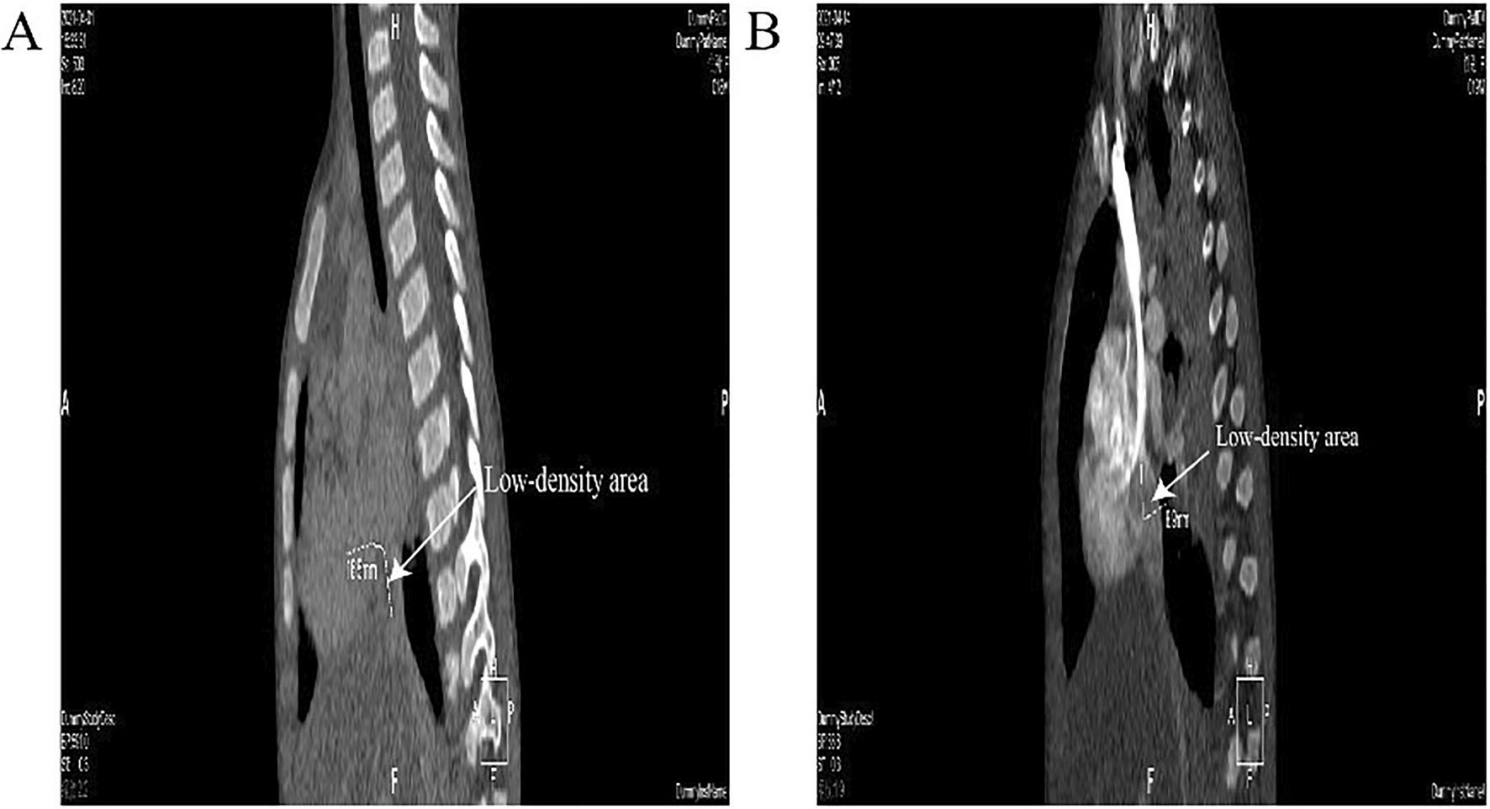
CTA images during the patient's course of disease. (A) The CTA image of April 1 shows the presence of low‐density area. (B) The CTA image of April 14 shows the presence of low‐density area in the right atrium. CTA, computed tomography angiography.

Furthermore, diagnostic evaluation included a 3D reconstruction of the thoracic great vessels using CT imaging on April 1, which unveiled a hypointense shadow within the proximal end of the inferior vena cava and the right atrium, suggestive of a thrombus. Specific images illustrating these findings were shown in Figures 1, 2, 3, 4.

The patient underwent a symptomatic therapeutic regimen comprising anti‐infective agents, alongside anticoagulation therapy which included sodium heparin (low molecular weight heparin calcium administrated via subcutaneous injection for a duration of 2 weeks) and warfarin (administrated for 19 days). The cTTE performed on April 13 revealed no noteworthy filling defect within the right atrial cavity. A subsequent computed tomography angiography (CTA) conducted on April 14 exhibited a hypointense shadow in the right atrium, interrupted as a thrombus, and a heterogeneous density observed within the proximal segment of the inferior vena cava. An echocardiogram on April 21 did not delineate any significant anomalies. Following a treatment course of 5 weeks, the patient manifested notable improvement in the symptoms of rash and diarrhea and was consequently discharged.

## 3 DISCUSSION

In this clinical case, our initial diagnostic consideration revolved around the possibility of sepsis‐induced thrombosis. This hypothesis arose due to the patient's conspicuous presentation featuring a profound systemic inflammatory response, typified by an elevated fever exceeding 38 degrees Celsius and a peripheral blood leukocyte count of 20.3 × 109/L, concurrent with the presence of pneumonia. Sepsis frequently cooccurs with disseminated intravascular coagulation (DIC), a condition characterized by widespread microthrombosis, and patients experiencing sepsis conjunction with DIC are known to exhibit elevated mortality rates. It is noteworthy that the main feature of sepsis‐associated DIC is the formation of microthrombi, while thromboembolic events affecting large arteries and veins are comparatively less common.^3^

Consequently, to conclusively ascertain whether the patient was afflicted with sepsis, we conducted bacterial blood cultures. The outcomes of these blood culture analyses unequivocally negated the presence of septic infection in the patient.

DIHS, also referred as drug reaction with eosinophilia and systemic symptoms, represents a potentially life‐threatening multiorgan hypersensitivity reaction. This syndrome typically manifests as a maculopapular, measles‐like rash accompanied by a fever exceeding 38°C, which emerges abruptly 2–3 weeks post drug initiation.^4^ Associated complications of DIHS include myocarditis, Pneumocystis jirovecii pneumonia, sepsis, and gastrointestinal hemorrhage, among others.^5^

We posit that the placement of an internal jugular vein catheter during the child's hospitalization at the local hospital may have triggered endothelial injury, resulting in diminished blood flow. Subsequently, the DIHS could have acted as a catalyst in fostering thrombus formation. The intestinal complications associated with DIHS likely contributed to severe diarrhea and dehydration, exacerbating blood viscosity. Notably, the child displayed elevated platelet levels during hospitalization in our institution (361 × 10^9^/L), a circumstance highly predisposing to abnormal platelet aggregation, a recognized contributing factor to thrombotic events.^6^, ^7^ Regrettably, in the current case, an assessment of the child's thrombophilia genes was not implemented. Thromboembolic events in pediatric patients are relatively rare, with the main risk factors encompassing malignancy, autoimmune disorders, congenital heart anomalies, central venous catheterization, and hereditary thrombophilia conditions.^8^ Of particular concern, the placement of central venous catheters carries a significant risk, with right arterial thrombosis emerging as a prominent complication.^9^ The risk of right atrial thrombosis is two‐fold: (1) the thrombus may traverse between the atria and ventricles with the diastolic contraction of the heart and can become lodged in the tricuspid valve orifice. This critical scenario gives rise to mechanical impediment, and in its most dire manifestation, precipitates sudden cardiac fatality; (2) secondly, an equally formidable facet of this risk profile resides in the propensity of the thrombus to dislodge. Such an eventuality poses an immediate and hazardous threat, as the liberated thrombus embarks on a treacherous journey through the pulmonary circulation, culminating in a catastrophic and potentially fatal event—a massive pulmonary embolism.^10^ Hence, prompt diagnosis and intervention assume paramount importance in such cases.

Cardiac space‐occupying lesions are a common occurrence in clinical practice, encompassing a diverse spectrum of diseases, including thrombosis, benign tumors, primary malignancies, and secondary malignancies.^11^ Clinical presentations vary depending on the specific disease, often lacking specificity. Distinguishing these lesions accurately is pivotal for selecting appropriate treatment strategies and enhancing patient outcomes.

Among various imaging modalities, echocardiography emerges as the preferred choice due to its convenience, rapidity, absence of radiation, ease of follow‐up, and excellent soft tissue resolution.^12^ TTE enables the dynamic, real‐time visualization of space‐occupying lesions, providing critical insights into their dimensions, anatomical localization, and morphological characteristics, along with the associated hematological alterations. However, TTE may be influenced by various factors, including patient‐specific factors such as obesity, chest wall configuration, and the presence of excessive air within the pulmonary parenchyma.^13^, ^14^ In contrast, transesophageal echocardiography (TEE) offers superior acoustic access and image quality.^15^, ^16^ Nonetheless, TEE carries the risk of complications such as oropharyngeal injury, dysphagia, gastroesophageal perforation, gastrointestinal bleeding, and even cardiac arrhythmias, which can have significant mortality implications.^17^, ^18^, ^19^, ^20^, ^21^, ^22^ While CT scanning is rapid, its ability to differentiate between benign and malignant lesions is limited, and it exposes patients to higher radiation levels than other imaging techniques. Magnetic resonance imaging (MRI) yields highly detailed images and can capture subtle changes often missed by other imaging techniques. However, MRI is costly and requires patients to remain immobile for extended periods, potentially causing discomfort. Contrast‐enhanced echocardiography is performed by injecting a contrast agent into the peripheral vessels to create an acoustic image in the cardiac chambers and myocardial tissue. This advanced imaging technique not only augments the visual clarity when assessing intracardiac space‐occupying lesions but also enables the evaluation of lesion perfusion through the intensity of the recorded image. Consequently, this dual capability assists in the refined characterization of the lesion's nature.^23^ Furthermore, cTTE possesses several noteworthy merits, including its inherent safety, noninvasiveness, and independence from variations in blood flow rates. In addition, cTTE permits real‐time visualization, enabling the immediate assessment of shunt conditions and hemodynamic parameters, providing valuable clinical insights.^24^

These microbubbles are used as a contrast agent, and because they are filtered and destroyed by the pulmonary circulation, they are selectively imaged in the right cardiac system, with no contrast reflection in the left cardiac system unless RLS is present.^25^, ^26^ cTTE is a technique employed to enhance the visualization of the right heart system during echocardiographic imaging. This approach entails the intravenous injection of an acoustic contrast agent, typically consisting of microbubbles with a diameter exceeding 10 microns. These microbubbles serve as contrast agents and are selectively imaged within the right heart system, owing to their filtration and destruction by the pulmonary circulation—unless a right to left shunt exists.^25^ In addition to the observation of shunted heart disease, right heart system imaging is also useful for endocardial and intracardiac occupancy of the right heart cavity in poorly transmissive conditions, and the filling defect of the reflected signal of the microbubbles suggests occupancy. The filling defect of the microbubble reflection signal suggests an occupying lesion reading, so it can increase the detection rate of tumors in the right heart system.

The selection of a suitable solution for enhancing right heart acoustic contrast is a critical aspect of clinical practice and varies based on the physician's experience. Choices typically encompass solutions such as 5%–50% glucose solution, saline, indocyanine blue‐green, hydrogen peroxide solution, sodium bicarbonate solution, and autologous blood among others. These options offer varying degrees of contrast enhancement, but a comprehensive and systematic comparative analysis is currently lacking in the literature. Carbon dioxide microbubbles are now often produced by the acid‐base reaction between sodium bicarbonate and vitamin C to produce carbon dioxide, or by mixing sodium bicarbonate with acetic acid to produce carbon dioxide, which produces bubbles that are larger, unevenly observed by the naked eye and enter the right heart with an uneven distribution of echogenicity, coarse echogenicity, and short duration.^26^ Hydrogen peroxide solution was widely used by clinicians in the past. However, now it has been abandoned due to its toxic side effects.^27^

At present, the preparation for right heart contrast echocardiography is still widely used in clinical practice, which is hand‐vibrated normal saline air microbubbles to increase the effect of right heart contrast.^28^ Both hand‐agitated glucose solution and hand‐agitated saline solution with blood had good imaging effects.^29^, ^30^, ^31^ However, the stirred saline with blood has a higher sensitivity, but the improved method of adding 1 mL of blood to the patient is not acceptable to every patient and increases the unknown risk.^32^, ^33^

The patient underwent three cTTEs during the course of the disease. On March 31, a prominent space‐occupying lesion became evident at the entrance of the inferior vena cava into the right atrium during diagnostic evaluation. Following a brief interval of 2–3 cardiac cycles, right atrial contrast enhancement was undertaken, revealing a filling defect measuring approximately 26.0 × 9.3 mm within the right atrial cavity. A subsequent cTTE, performed on April 6, did not demonstrate significant alterations in the previously observed findings. Commencing on April 6, the patient commenced anticoagulation therapy with warfarin and sodium heparin. Subsequently, on April 13, a repeat cTTE was conducted. Following the intracardiac injection of a hand‐vibrated dextrose solution over three cardiac cycles, the examination revealed no substantial filling defect within the right atrial cavity. During the course of anticoagulation therapy, the patient remained free from symptoms indicative of pulmonary embolism, such as dyspnea and chest pain, as well as manifestations of cerebral embolism, including limb numbness and speech disorders. This indicates the successful dissolution of the thrombus within the inferior vena cava without any evidence of thrombus dislodgement. However, a subsequent repeat of cardiac CTA on April 14, 2021, still suggested the presence of a discernible low‐density area within the right atrium, which raised suspicion of a possible residual thrombus. Additionally, the density within the proximal portion of the inferior vena cava exhibited nonuniform characteristics. Notably, these findings from CTA appeared to be at odds with the earlier echocardiography and cTTE assessments, both of which indicated the successful dissolution of the thrombus within the inferior vena cava. This discrepancy underscores the potential superiority of cTTE as a diagnostic modality for this specific pathology compared to CTA.

As early as 2014, the American Society of Echocardiography mentioned that a hand‐vibrated saline contrast agent is helpful in assessing RLS and detecting residual shunt after defect closure, and that hand‐vibrated sterile saline contrast without crossing the pulmonary circulation is the preferred method to screen for PFO.^34^ Over time, as contrast agents continued to evolve, reports have surfaced regarding the efficacy of hand‐vibrated 50% glucose as a contrast agent. These reports have demonstrated promising results in both animal experimentation and clinical application. In our current study, we employ a hand‐vibrated glucose solution, which harnesses manual vibration to generate microbubbles for enhancing right heart contrast. This research initiative has undergone rigorous scrutiny, including ethical and technical evaluations, and is presently underway at the Children's Hospital of Chongqing Medical University. We conducted comprehensive assessments of blood glucose levels, liver function, and kidney function both before and after cTTE. Importantly, our findings indicate that there were no significant alternative changes in any of the above tests before and after the imaging. This affirms the safety and reliability of employing a hand‐vibrated glucose solution for cTTE applications.

The cTTE test is indeed a safe, efficacious, and replicable diagnostic tool, with distinct advantages in pediatric patients as compared to adults. Notably, following the administration of a hand‐vibrated glucose solution as a contrast agent, pediatric subjects achieve visualization within a remarkably swift 2–3 cardiac cycles, whereas adult counterparts necessitate more extended 8 cardiac cycles for comparable outcomes. The cTTE provides an important basis for clinical interventions and preoperative and postoperative evaluation. Moreover, cTTE plays a pivotal role in the precise localization and quantification of thrombi and foreign bodies within the vicinity of the right heart, boasting a commendable accuracy rate in this regard.

## 4 CONCLUSION

This case report underscores the substantial clinical utility of cTTE. Due to the advantages of a long duration of visualization and safety, cTTE is a reliable and feasible method for the diagnosis and follow‐up of diseases presenting with RLS.

## AUTHOR CONTRIBUTIONS

Hui‐ru Zhu wrote article. Xiaojuan Ji and Xu Zhu discussed the case together and reviewed the article as senior authors. Ting‐ting Ran provided information on the case. All authors contributed to the article and approved the submitted version.

## CONFLICT OF INTEREST STATEMENT

The authors declare no conflict of interest.

## ETHICS STATEMENT

This study involving human participants was reviewed and approved by the Ethics Committee of Children's Hospital of Chongqing Medical University (056/2020). The patients' parents have provided their written informed consent to participate in this study. Written informed consent was obtained from the patients' parents for the publication of any potentially identifiable images or data included in this article. Informed written consent was obtained from the patients' parents for publication of this report and any accompanying images.

## Data Availability

The data that support the findings of this study are available from the corresponding author upon reasonable request.
